# An Improved Stress-Scale Specifically Designed to Measure Stress of Women with Newly Diagnosed Breast Cancer

**DOI:** 10.3390/ijerph18052346

**Published:** 2021-02-27

**Authors:** Tso-Ying Lee, Shih-Chun Hsing, Chin-Ching Li

**Affiliations:** 1 Department of Nursing, Cheng Hsin General Hospital, Taipei 112, Taiwan; ch4006@chgh.org.tw; 2 School of Nursing, National Taipei University of Nursing and Health Sciences, Taipei 112303, Taiwan; 3 Center for Healthcare Quality Management, Cheng Hsin General Hospital, Taipei 112, Taiwan; ch8363@chgh.org.tw; 4 Department of Health Care Management, National Taipei University of Nursing and Health Sciences, Taipei 112303, Taiwan; 5 Department of Nursing, Mackay Medical College, New Taipei City 25245, Taiwan

**Keywords:** stress scale, breast cancer, confirmatory factor analysis, exploratory factor analysis, NDBCSS

## Abstract

Most breast cancer patients are middle-aged women actively involved in establishing a family, developing a career, or raising children. With the exception of the Newly Diagnosed Breast Cancer Stress Scale (NDBCSS), few stress scales have been designed for women with breast cancer. This study checked the dimensionality of the NDBCSS by confirmatory factor analysis (CFA) and the results showed a poor fit, indicating an urgent need for improvement. Exploratory factor analysis (EFA) using the varimax rotation method was performed to improve the model, the revised NDBCSS (NDBCSS-R), which showed a good Kaiser-Meyer-Olkin value, Bartlett’s test of sphericity, and internal consistency reliability. The NDBCSS-R showed improved indices compared with NDBCSS, including: chi-square fit statistics/degree of freedom (CMIN/DF), goodness-of-fit index (GFI), adjusted goodness of fit index (AGFI), normed fix index (NFI), relative fit index (RFI), incremental fix index (IFI), Tucker–Lewis index (TLI), comparative fix index (CFI), root mean square error of approximation (RMSEA), root mean square residual (RMR), parsimonious goodness-fit-index (PGFI), and parsimonious normed fit index (PNFI). In conclusion, the improved NDBCSS-R can provide health professionals with an early understanding of the stress levels of women with breast cancer so that they can provide immediate medical intervention to prevent vicious cycles in a timely manner.

## 1. Introduction

In recent years, the prevalence and incidence of breast cancer has continued to rise. Breast cancer is currently the most common cause of cancer death in eleven regions of the world [1,2]. Among the top ten cancers, the incidence and mortality of breast cancer patients worldwide is 24.2% and 15.0%, respectively [2,3]. Moreover, the incidence of breast cancer varies widely among countries. According to data from the World Health Organization (WHO), the median age of newly diagnosed breast cancer in black women and white women is about 63 years [4]. However, in Asia, women diagnosed with breast cancer are relatively young (Taiwan, 45–54 years; Korea, 40–49 years; India, 50–54 years) [5,6,7]. Many studies have found that early detection of breast cancer can increase cure rates and five-year survival rates by up to 90%. Many countries have developed medical policies to offer early breast cancer screening services to high-risk women [8]. For example, in Taiwan and South Korea, patients aged 45–69, and patients aged 40–44 with a family history of breast cancer, are given a mammographic x-ray (MMx) subsidy every two years [5,6].

Although the development of cancer research has made great progress and the cure rate of cancer patients has improved [9], the health problems of patients with breast cancer still deeply affect the entire social structure of a patient. Women with breast cancer often must deal with changes in their quality of life and problems caused by their disease symptoms. Significantly, most patients with breast cancer are middle-aged women who are at the life stage when they are establishing a family, developing a career, raising children or a combination of these activities. 

Negative changes in body image are often part of the side effects of treatment. This is especially significant among women whose body image centers around their femininity. Previous studies have found that following breast surgery, women have depression and anxiety associated with low levels of self-esteem and psychological coping strategies [10,11]. In addition, several studies have found that breast cancer patients experience high levels of anxiety at the beginning of treatment, with the anxiety level relieved temporarily, but then quickly increasing [12,13]. Current theory generally holds that a patient’s first encounter with cancer may cause increased anxiety. Subsequently, although patients try to adapt to their cancer and attempt to reduce their anxiety level, therapy (chemotherapy, radiotherapy and prolonged treatment) may further greatly increase depression and anxiety levels due to a decline in quality of life. As a result, breast cancer can seriously affect a woman’s identity, body image, self-esteem and cause very significant changes to socioeconomic and interpersonal relationships, eventually leading to psychological stress in these breast cancer patients [3,14,15,16,17]. The resulting long-term stress may lead to physical problems, such as hypertension, as well as mental health problems, such as anxiety and depression [18,19,20]. Additionally, some studies have demonstrated that breast cancer patients under the age of 50 suffer a greater psychological burden than older patients, and that the psychological burden does not improve over time [21,22]. 

Of great concern is the vicious cycle created, that is, psychological problems after treatment gradually affect the patient’s family and work conditions, and then expand, in turn resulting in additional physical and mental burdens [23,24]. We submit that if a patient’s stress index and related information can be identified early in the diagnosis and treatment of breast cancer, a more informed intervention by health professionals might help to reduce the patient’s stress and prevent this vicious cycle in a timely manner.

A stress scale is a tool widely used by health professionals to detect the stress levels of patients. In the past, perceived stress scales were developed to detect different aspects of stress and target different diseases. Universal stress scales have often been used to measure cancer-related stress, such as the following: the Davidson Trauma scale (DTS); Stress Appraisal Measure (SAM); Impact of Event scale (IES); Center for Epidemiological Studies Depression scale (CES-D); and the Perceived Stress Scale (PSS). Among them, PSS is the most widely used to examine or evaluate stress caused by events, physical and mental diseases, and the effectiveness of subsequent stress management programs [25,26,27]. These universal stress scales are either psychiatric or unspecific instruments. The application of such instruments to cancer patients does not fully address the subjective aspect of the health of cancer patients. For example, most of the questions asked of cancer patients are items related to psychiatric symptoms, but are not related to the specific needs and distress of the disease. If the psychological findings are inconsistent with those of psychiatric disorder, but the patient still shows high subjective distress and needs psychosocial support, this will result in a distorted picture [28]. For example, among women who have just started a family or are raising children, the reality of a breast cancer diagnosis may make these women worry about the impact on the family. Moreover, the results provided by disease-specific questionnaires can more adequately reflect a patient’s experience because they can more clearly pinpoint the further support required as a consequence of therapeutic (or psycho-oncological) treatments [29]. These factors strongly support the development of a stress scale for women-specific diseases, such as breast cancer.

Chinese people tend to communicate their emotional problems by developing psychosomatic symptoms, rather than openly admitting their feelings [30,31]. As with other cancers, when a patient first faces a diagnosis of breast cancer, the patient is under tremendous pressure. A study by Chuang and Chin (2002) showed that women with newly diagnosed breast cancer face three major issues: insufficient information acquisition, loss of self-control, and an increased sense of uncertainty [32]. Insufficient information acquisition involves patients feeling that they cannot fully obtain sufficient information about breast cancer, including medical treatment and medical care. Insufficient self-control refers to things that cannot be carried out in accordance with the predetermined plan, resulting in the inability to plan the next step (e.g., threat of survival). Increased uncertainty refers to the vagueness of events related to the patient’s breast cancer. For example, will the events that happen to other breast cancer women, such as divorce, happen to me? The fear that comes from experiencing sudden and unexpected cancer, the heavy financial and emotional burden, and indirect ways of expressing emotion, may cause additional serious psychological trauma. Therefore, it would be advantageous to patients, their families and clinical staff to measure and utilize the stress index, and related information, of patients with breast cancer in order to facilitate timely intervention.

The theoretical definitions used in our initial questionnaire were developed from the content of qualitative interviews with 20 women in Taiwan, all with newly diagnosed breast cancer, based upon their interviews with three medical experts. Since two of the interviewing medical experts had been diagnosed with breast cancer previously, they were viewed as better able to establish a relationship with the patients and to more fully understand their reactions to the presence of stressors, emotional and physiological reactions, and changes in their living environment. The initial stress questionnaire was developed based on both the interview data and relevant literature. Subsequently, 125 women newly diagnosed with breast cancer were further tested using this stress questionnaire. The PSS and the Hospital Anxiety and Depression Scale (HADS) were used to determine the degree of stress and stress-related symptoms of anxiety and depression, which now constitutes the prototype of the Newly Diagnosed Breast Cancer Stress Scale (NDBCSS) for breast cancer patients [33]. Although the scale has been proven to have acceptable reliability and validity in China [34] and Greece [35], we found that the scale still has poor model fit. In this study, we recruited a group of 195 women with newly diagnosed breast cancer and used factor analysis to improve the structure of the NDBCSS for breast cancer patients.

## 2. Materials and Methods

### 2.1. Original NDBCSS

The original NDBCSS scale, with four-factor structure and 17 items, was established in 2013 to measure the stress perceptions of newly diagnosed breast cancer women, with a Cronbach’s α = 0.84. The scale appears to have sufficient psychometric properties to be used as a designated measure of perceived stress in women newly diagnosed with breast cancer. The NDBCSS scale consisted of four main factors, each with 4 to 5 items. These factors include: unpredictable perceptions (5 items); uncontrollable perceptions (4 items); heavy psychological load perceptions (4 items); and facing challenge perceptions (4 items). The scale is scored as follows: strongly agree = 4; agree = 3; somehow agree = 2; disagree =1. A higher score indicates greater stress.

### 2.2. Participants

A total of 195 participants were recruited from a breast surgery clinic at the Medical Center of Tri-Service General Hospital in Taiwan, and all participants were citizens of Taiwan. This study was approved by the Institutional Review Board. Inclusion criteria for participation were patients with breast cancer newly diagnosed by a physician, who had agreed to surgery and to participate in this study, and had signed informed consent. The time from diagnosis to completion of the questionnaire did not exceed two weeks. The day before the patient underwent breast cancer surgery, the researchers explained the study protocol and asked the patient to complete the NDBCSS and to provide personal demographic information. In addition, participants were informed that the purpose of this study was to improve a tool for measuring the stress of the breast cancer patients, that the participant may withdraw from the study at any time, and that all data collected from patients will be kept confidential.

### 2.3. Statistical Analyses

In order to improve the original NDBCSS scale, a confirmatory factor analysis (CFA) was conducted to validate the original NDBCSS using a dataset from 195 newly recruited patients. A series of indices was used to evaluate whether the hypothesized hierarchical model exactly fits the data, including the chi-square fit statistics/degree of freedom (CMIN/DF), goodness-of-fit index (GFI), adjusted goodness of fit index (AGFI), normed fix index (NFI), relative fit index (RFI), incremental fix index (IFI), Tucker-Lewis index (TLI), comparative fix index (CFI), root mean square error of approximation (RMSEA), root mean square residual (RMR), parsimonious goodness-fit-index (PGFI), and parsimonious normed fit index (PNFI). All analyses were performed with the statistical Product and Service Solutions (SPSS) and Analysis of Moment Structures (AMOS) software. Good indicators were considered to be: CMIN/DF between 2–3; GFI > 0.85; AGFI > 0.80; NFI > 0.80; RFI > 0.80; IFI > 0.90; TLI > 0.90; CFI > 0.90; RMSEA < 0.08; RMR < 0.05; PGFI > 0.50; and PNFI > 0.50. 

Due to the poor fit of the original model, exploratory factor analysis (EFA) was further performed to examine the dimensionality of the original 17-item NDBCSS. First, Bartlett’s test of Sphericity and the Kaiser-Meyer-Olkin (KMO) were performed to evaluate the suitability of data that were to be used for Factor Analysis. Second, scree plot was created to further identify the optimal number of factor solution. Subsequently, the principal components method with varimax rotation was used for factor extraction, and CFA was performed to examine how well the newly included NDBCSS items represent the constructs extracted by EFA. In addition, a second-order factor was created to represent the general concept of stress, which may be explained by factors extracted from EFA. All indices of the new model were determined and compared with that of the original model. Finally, correlation analysis was used to explore the correlation between different factor structures.

## 3. Results

### 3.1. Clinical and Demographic Characteristics

In order to evaluate whether or not the original NDBCSS is suitable for breast cancer patients today, 195 patients with breast cancer were recruited for the study. Table 1 shows the clinical and demographic characteristics of all participants. The mean age at diagnosis was 52.2 years, ranging from 28 to 89 years. Of the 195 subjects, 49 participants (25.13%) were aged < 45 years, 80 (41%) aged 45–54 years, 45 (23.1%) aged 55–64 years, and 18 (9.2%) aged ≥ 65 years. Most participants were married (80%), and 43.1% of the participants had a college degree or above. Nearly half of the participants (54.17%) were still in employment. The pathology reports showed that most participants were diagnosed in Stage 0–2 with 58 (33.72%) participants in Stage 0, 63 (36.63) in Stage 1, and 45 (26.16%) in Stage 2. Six patients (3.49%) were diagnosed in Stages 3–4. Most of the participants (80.42%) had no family history of breast cancer, and 38.1% had received regular mammography. All participants underwent a Self-Awareness of Health examination, and the results showed that 74.6% of participants worried about their health problems.

### 3.2. Confirmatory Factor Analysis of the Original Four-Factor Structure of the NDBCSS

Confirmatory factor analysis (CFA) was conducted to examine the dimensionality of the original NDBCSS, a four-factor model with 17 items, and to determine whether the original model adequately fit the newly collected data. After CFA analysis of 195 newly recruited breast cancer patients, the results showed a poor fit for the original model. All the important indices did not meet the standard fitting criteria of model fitness, including: CMIN/DF = 3.30; goodness of fit index (GFI = 0.80); the adjusted goodness of fit index (AGFI = 0.73); normed fit index (NFI = 0.72); relative fit index (RFI = 0.66); incremental fit index (IFI = 0.79); Tucker–Lewis index (TLI = 0.74); comparative fit index (CFI = 0.78); the root-mean-square error of approximation (RMSEA = 0.11); root mean square residual (RMR = 0.06); parsimonious goodness-fit-index (PGFI = 0.59); and the parsimonious normed fit index (PNFI = 0.60). All indices suggested that the original model did not adequately fit the data from the breast cancer patients, and this model needed to be revised and improved.

### 3.3. Model Improvement of the NDBCSS by Exploratory Factor Analyses

**Figure 1:**
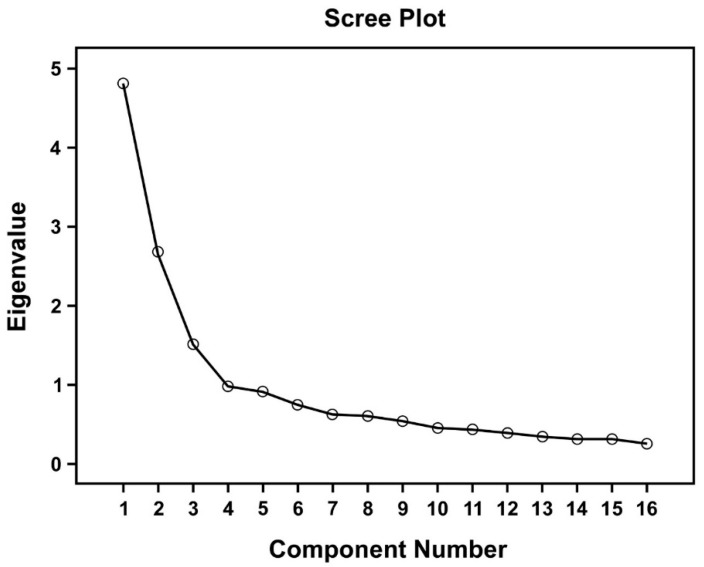
Scree plot of NDBCSS in factor analysis with extraction method.

In order to improve the original NDBCSS, an exploratory factor analysis (EFAs) was conducted. The result of scree plot indicated that the four-factor solution was the best solution (Figure 1). The curve in scree plot levelled off after the first four components, with eigenvalues of 4.83, 2.69, 1.52 and 0.99, respectively. We further examined the three- and five-factor solutions. The five-factor solution was discarded because factor 1 and 2 included most of the items, resulting in only one item being in factor 5. The three-factor solution was also rejected because two items were cross-loaded in different factors. In addition, the three-factor solution did not meet the standard fitting criteria of model fitness. Thus, the four-factor solution was supported as the best model and was selected for following analysis. After examining the relationship between each item, item 5 (illness makes me worry about my job) was dropped because of low commonalities with other items. Finally, a new Revised NDBCSS (NDBCSS-R) was generated using a four-factor model containing a total of 16 rearranged items, each factor containing 3–5 items. Table 2 showed the new NDBCSS-R has good internal consistency reliability (Cronbach’s α = 0.84). Subsequently, the NDBCSS-R underwent EFA analysis using the varimax rotation method and the results of factor matrix of NDBCSS-R after varimax rotation is shown in Table 3. The Kaiser-Meyer-Olkin (KMO) value was 0.83, and the Bartlett’s test of sphericity was statistically significant (*p* < 0.001). These indicated that these variables were suitable for subsequent factor analysis.

### 3.4. Confirmatory Factor Analysis of the New NDBCSS-R

**Figure 2:**
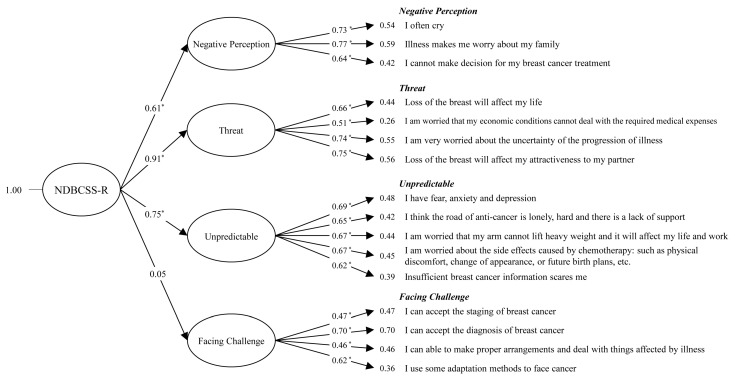
Four-factor model of the NDBCSS-R stress scale with factor loadings and factor covariance. Asterisks indicate statistical significance at *p* < 0.05.

CFA was then further used to examine whether or not the new NDBCSS-R had good model fit. As shown in Table 4, the new NDBCSS-R has a good fit index after model modification: CMIN/DF = 2.48; GFI = 0.92; AGFI = 0.81; NFI = 0.8; RFI = 0.83; IFI = 0.92; TLI = 0.90; CFI = 0.92; RMSEA = 0.08; RMR = 0.04; PGFI = 0.63, and; PNFI = 0.65. Although the value of RMSEA was not less than 0.08, the value 0.084 was very close and was accepted. It is worth noting that all other important indices meet the standard fitting criteria of model fitness [36,37,38,39,40,41,42,43,44,45,46], indicating that the revised model has been greatly improved compared to the original model. The standardized factor loadings in CFA are displayed in Figure 2. The specified structural model contains four factors, including “Negative perception”, “Threat”, “Unpredictable”, and “Facing Challenge”. The factor loadings of these subscales ranged from 0.64 to 0.77 for the “Negative perception” subscale, 0.51 to 0.75 for the “Threat” subscale 0.62 to 0.69 for the “Unpredictable” subscale, and 0.46 to 0.70 for the “Facing challenge” subscale.

The prototype NDBCSS included “Unpredictable”, “Uncontrollable”, “Heavy psychological load”, and “Facing challenge”. In the revised NDBCSS-R, Factor 1 is renamed “Negative perception” (was “unpredictable”) and consists of S01 (I often cry), S02 (Illness makes me worry about my family), and S06 (I cannot make a decision for my breast cancer treatment). Factor 2 is renamed “Threat” (was “uncontrollable”) and consists of item S03 (Loss of the breast will affect my life), S09 (I am worried that my economic conditions cannot deal with the required medical expenses), S10 (I am very worried about the uncertainty of the progression of illness), and S12 (Loss of the breast will affect my attractiveness to my partner). Factor 3 is still “Unpredictable”, but the items were reorganized to include item S04 (I have fear, anxiety and depression), S07 (I think the road of anti-cancer is lonely, hard and there is a lack of support) S8 (I am worried that my arm cannot lift a heavy weight and it will affect my life and work), S11 (I am worried about the side effects caused by chemotherapy: such as physical discomfort, change of appearance, or future birth plans, etc.), and S13 (Insufficient breast cancer information scares me). The composition of factor 4 was not changed, and is still called “Facing challenge”, including S15 (I can accept the diagnosis of breast cancer), S16 (I can able to make proper arrangements and deal with things affected by illness), and S17 (I use some adaptation methods to face cancer). In the improved NDBCSS-R, individual scores on the NDBCSS-R scale can range from 0 to 64 with higher scores indicating higher stress.

### 3.5. Analysis of the Inter-Relationship between the Four-Factor Structures

Table 5 shows the inter-factor correlations between the four factors of the new NDBCSS-R. After removing item 5, the inter-factor correlation of the four factors ranged from 0.01 to 0.62, suggesting satisfactory discrimination. The correlation between “Negative perception” and “Threat”, “Negative perception” and “Unpredictable”, and “Threat” and “Unpredictable” were 0.50, 0.36, and 0.62, respectively (*p* < 0.001). The correlations between “Facing Challenge” and the other three factors were extremely low, ranging from 0.01 to 0.03.

## 4. Discussion

The prototype NDBCSS was designed to measure stress perceptions in newly diagnosed breast cancer patients. However, CFA analysis indicated that this prototype had a poor model fit. To improve this scale, EFA analysis was conducted and found that the low-commonality of item 5 (Illness makes me worry about my job) suggested it should be removed and the remaining 16 items should be rearranged. The results of the scree plot, the values of factor loadings, and the associated confidence interval data showed that three- and five-factor solutions were unstable or had poor fit. Thus, the four-factor solution is still the best model and a new, revised NDBCSS-R was generated (Supplementary Materials). The new NDBCSS-R does have a better fit index than the old one (Table 4) and also has a high level of internal consistency. This evidence provided robust support for NDBCSS-R.

Women with breast cancer experience a range of negative reactions after diagnosis, including anger, depression, fear of treatment, psychological distress and despair. However, very few stress scales have been specifically developed for women with breast cancer. Therefore, many studies still use a universal stress scale to determine the stress index in cancer patients, such as the most commonly used PSS [47], DTS [48], and SAM [4]. The global measure of perceived stress, PSS, was established by Sheldon Cohen et al. [49] in 1983 to evaluate stress deriving from ongoing life circumstances. The PSS was developed using two groups of college students and one group of participants in a community smoking-cessation program, but did not include cancer patients. Thus, as the PSS was not designed for any specific population, it is a more comprehensive and versatile pressure scale. Conversely, the PSS may be less sensitive to stress perception in specific populations, for example, cancer populations.

The DTS is a self-reporting measure of post-traumatic stress disorder (PTSD) symptoms and is commonly used for large-scale evaluations of post-traumatic stress, such as veterans with PTSD [50]. The SAM includes major stress assessments, secondary stress assessments (e.g., coping resources), and overall perceived stressfulness, such as appraisal and coping with depressive symptoms in both stroke patients and spouses [51]. These scales are indeed suitable for children, adults and the general public of all ages but do not consider the psychological pressures faced by cancer patients, especially women with breast cancer who are starting families and parenting.

Breast cancer patients face severe psychological and physical reactions both before and after treatment. In addition to persistent pain, injuries to the arm and shoulder, and persistent high fatigue, breast cancer patients may experience psychological reactions, including psychological distress, depression, and anxiety [52,53,54,55,56]. A randomized pilot study showed that the degree of anxiety, depression and psychological distress of newly diagnosed breast cancer patients can be improved through individually tailored nurse-navigation interventions [57].

Another descriptive-exploratory study also showed that nurse navigator home visit interventions can successfully reduce the fear and distress among patients with a new breast cancer diagnosis [58]. In addition, nursing interventions have been shown to increase the level of hope in cancer patients through promoting psychological wellbeing and reducing psychological problems [59]. Therefore, it becomes very important to determine, in a timely manner, and understand the stress level of newly diagnosed breast cancer patients through a dedicated stress scale. Once the stress level of newly diagnosed breast cancer patients is understood, healthcare providers and their family members can not only help patients to get rid of negative emotions and fatalistic beliefs, but they can also learn to actively respond to cancer treatment and to plan their future life, achieving the goal of eliminating the vicious stress-illness-stress cycle often caused by breast cancer at the early stages.

Several limitations should be noted in this study. This stress scale was evaluated in Taiwan in a population of Taiwanese women with newly diagnosed breast cancer. The general conclusions of this study may therefore only be applicable to similar populations. Breast cancer patients from different countries and cultures may face different pressures, so this scale may need to be appropriately modified according to different cultural sensitivities. Compared with other CFA studies, the sample size of this study was relatively small. Since EFA and CFA were conducted using the same population, it may be necessary to conduct new research in order to adjust and verify the content items and confirm the new factor structure so as to ensure applicability to other populations.

## 5. Conclusions

As breast cancer survival rates have gradually improved, clinicians and researchers are now focusing more on improving quality of life and patient-centric survival outcomes. In order to provide newly diagnosed breast cancer patients with better practical strategies for maintaining hope and overcoming distress, depression, anxiety and fear of treatment, a better understanding of stress levels will be needed. In alignment with this new perspective, the improved NDBCSS-R has greater psychometric capability than the original NDBCSS, enabling the NDBCSS-R to better detect the stress level of breast cancer patients. In addition, the NDBCSS-R shows satisfactory reliability, validity and sufficient psychometric properties to specifically measure the perceived stress level of newly diagnosed breast cancer patients. These advances will allow the new NDBCSS-R scale to facilitate more precise clinical interventions so as to relieve patient stress and help promote patient motivation for treatment.

## Figures and Tables

**Table 1:** Baseline demographic and clinical characteristics.

Variables	N = 195 (%)
Age	
<45	49 (25.13)
45–54	80 (41.00)
55–64	45 (23.10)
≥65	18 (9.20)
Marital status	
Unmarried (single, sivorced, separated and other)	39 (20.00)
Married	156 (80.00)
Education level	
High school and below	111 (56.96)
College and above	84 (43.10)
Income (NTD)	
<45,000	33 (25.00)
45,000–69,999	35 (26.52)
70,000–99,999	17 (12.88)
≥100,000	47 (35.61)
Employment	
No	104 (54.17)
Yes	88 (45.83)
Tumor stage	
0	58 (33.72)
1	63 (36.63)
2	45 (26.16)
3	4 (2.33)
4	2 (1.16)
Family history	
No	152 (80.42)
Yes	37 (19.58)
Mammographic x-ray	
No	117 (61.9)
Yes	72 (38.1)
Self-awareness of health	
Well	49 (25.39)
Not well	144 (74.61)

**Table 2:** Cronbach α of the original Newly Diagnosed Breast Cancer Stress Scale (NDBCSS) and improved NDBCSS-R.

Scales	Cronbach α				
	All	Factor 1	Factor 2	Factor 3	Factor 4
Original NDBCSS	0.82	0.76	0.65	0.76	0.79
Improved NDBCSS-R	0.84	0.75	0.76	0.79	0.79

**Table 3:** Rotated factor pattern matrix and interfactor correlations.

Item No.	Factor 1	Factor 2	Factor 3	Factor 4
	Negative Perception	Threat	Unpredictable	Facing Challenge
S01	**0.83**	0.11	0.15	−0.02
S02	**0.76**	0.38	−0.01	−0.12
S06	**0.72**	0.11	0.31	0.17
S03	0.27	**0.59**	0.32	−0.11
S09	0.32	**0.66**	0.00	0.33
S10	0.10	**0.75**	0.32	0.00
S12	0.20	**0.68**	0.33	−0.14
S04	0.23	0.11	**0.76**	−0.14
S07	−0.10	0.32	**0.55**	0.01
S08	0.07	0.30	**0.66**	0.00
S11	0.04	0.18	**0.75**	−0.06
S13	0.23	0.15	**0.69**	0.18
S14	−0.12	−0.06	−0.02	**0.72**
S15	−0.01	−0.03	0.00	**0.84**
S16	0.02	0.03	−0.03	**0.80**
S17	0.13	0.04	0.01	**0.76**

Bold items indicate major loadings for each item.

**Table 4:** The results of the confirmatory factor analysis.

Item	CMIN/DF	GFI	AGFI	NFI	RFI	IFI	TLI	CFI	RMSEA	RMR	PGFI	PNFI
Guideline	2–3	>0.85	>0.80	>0.80	>0.80	>0.90	>0.90	>0.90	<0.08	<0.05	>0.50	>0.50
Original (NDBCSS)	3.30	0.80	0.73	0.72	0.66	0.79	0.74	0.78	0.11	0.06	**0.59**	**0.60**
New (NDBCSS-R)	**2.48**	**0.92**	**0.81**	**0.87**	**0.83**	**0.92**	**0.90**	**0.92**	0.08	**0.04**	**0.63**	**0.65**

Notes. CMIN/DF = chi-square fit statistics/degree of freedom; GFI = goodness-of-fit index; AGFI = adjusted goodness of fit index; NFI = normed fix index; RFI = relative fit index; IFI = incremental fix index; TLI = Tucker-Lewis index; CFI = comparative fix index; RMSEA = root mean square error of approximation; RMR = root mean square residual; PGFI = parsimonious goodness-fit-index; PNFI = parsimonious normed fit index.

**Table 5:** Correlation coefficient of each dimension and main scale (N = 195).

	Negative Perception	Threat	Unpredictable	Facing Challenge
Negative perception	-	0.50 **	0.36 **	0.01
Threat		-	0.62 **	0.05
Unpredictable			-	0.03
Facing Challenge				-

** *p* < 0.001.

## Data Availability

The data and materials used in this study are available from the corresponding author on reasonable request.

## Supplementary Materials

The following are available online at https://www.mdpi.com/1660-4601/18/5/2346/s1, Table S1: Item scores of NDBCSS-R.

## References

[1] Banas T., Juszczyk G., Pitynski K., Nieweglowska D., Ludwin A., Czerw A. (2016). Incidence and mortality rates in breast, corpus uteri, and ovarian cancers in Poland (1980-2013): An analysis of population-based data in relation to socioeconomic changes. Oncotargets.

[2] Ferlay J., Colombet M., Soerjomataram I., Mathers C., Parkin D.M., Pineros M., Znaor A., Bray F. (2019). Estimating the global cancer incidence and mortality in 2018: GLOBOCAN sources and methods. Int. J. Cancer.

[3] Kirkham A.A., Beaudry R.I., Paterson D.I., Mackey J.R., Haykowsky M.J. (2019). Curing breast cancer and killing the heart: A novel model to explain elevated cardiovascular disease and mortality risk among women with early stage breast cancer. Prog. Cardiovasc. Dis..

[4] Cao W., Qi X., Cai D.A., Han X. (2018). Modeling posttraumatic growth among cancer patients: The roles of social support, appraisals, and adaptive coping. Psychooncology.

[5] Chen Y.P., Lu Y.W., Yang C.C. (2017). Breast cancer trend in Taiwan. MOJ Women’s Health.

[6] Choi S.W., Ryu S.Y., Han M.A., Park J. (2018). Higher breast cancer prevalence associated with higher socioeconomic status in the South Korean population; Has it resulted from overdiagnosis?. PLoS ONE.

[7] Mubarik S., Malik S.S., Wang Z., Li C., Fawad M., Yu C. (2019). Recent insights into breast cancer incidence trends among four Asian countries using age-period-cohort model. Cancer Manag. Res..

[8] Gibbons A., Groarke A., Sweeney K. (2016). Predicting general and cancer-related distress in women with newly diagnosed breast cancer. BMC Cancer.

[9] Kaplan H.G., Malmgren J.A., Atwood M.K., Calip G.S. (2015). Effect of treatment and mammography detection on breast cancer survival over time: 1990–2007. Cancer.

[10] Ando N., Iwamitsu Y., Kuranami M., Okazaki S., Nakatani Y., Yamamoto K., Watanabe M., Miyaoka H. (2011). Predictors of psychological distress after diagnosis in breast cancer patients and patients with benign breast problems. Psychosomatics.

[11] Ju H.B., Kang E.C., Jeon D.W., Kim T.H., Moon J.J., Kim S.J., Choi J.M., Jung D.U. (2018). Associations Among Plasma Stress Markers and Symptoms of Anxiety and Depression in Patients with Breast Cancer Following Surgery. Psychiatry Investig..

[12] Osborne R.H., Elsworth G.R., Hopper J.L. (2003). Age-specific norms and determinants of anxiety and depression in 731 women with breast cancer recruited through a population-based cancer registry. Eur. J. Cancer (Oxf. Engl. 1990).

[13] Hinz A., Krauss O., Hauss J.P., Höckel M., Kortmann R.D., Stolzenburg J.U., Schwarz R. (2010). Anxiety and depression in cancer patients compared with the general population. Eur. J. Cancer Care.

[14] Bertero C.M. (2002). Affected self-respect and self-value: The impact of breast cancer treatment on self-esteem and QoL. Psychooncology.

[15] Helms R.L., O’Hea E.L., Corso M. (2008). Body image issues in women with breast cancer. Psychol. Health Med..

[16] Hill J., Holcombe C., Clark L., Boothby M.R., Hincks A., Fisher J., Tufail S., Salmon P. (2011). Predictors of onset of depression and anxiety in the year after diagnosis of breast cancer. Psychol. Med..

[17] Piot-Ziegler C., Sassi M.L., Raffoul W., Delaloye J.F. (2010). Mastectomy, body deconstruction, and impact on identity: A qualitative study. Br. J. Health Psychol..

[18] Ciambella C.C., Taneja C., Dizon D.S., Wiggins D.L., Emmick C.M., Leonard K.L., Lopresti M.L., Witherby S., Cabral D., Snow S. (2019). Distress: Characterizing What Causes the Thermometer to Shift in Patients with Newly Diagnosed Breast Cancer Attending a Multidisciplinary Clinic. Ann. Surg. Oncol..

[19] Garofalo J.P., Choppala S., Hamann H.A., Gjerde J. (2009). Uncertainty during the transition from cancer patient to survivor. Cancer Nurs..

[20] Hoyer M., Johansson B., Nordin K., Bergkvist L., Ahlgren J., Lidin-Lindqvist A., Lambe M., Lampic C. (2011). Health-related quality of life among women with breast cancer—A population-based study. Acta Oncol. (Stock. Swed.).

[21] Howard-Anderson J., Ganz P.A., Bower J.E., Stanton A.L. (2012). Quality of life, fertility concerns, and behavioral health outcomes in younger breast cancer survivors: A systematic review. J. Natl. Cancer Inst..

[22] Kroenke C.H., Rosner B., Chen W.Y., Kawachi I., Colditz G.A., Holmes M.D. (2004). Functional impact of breast cancer by age at diagnosis. J. Clin. Oncol. Off. J. Am. Soc. Clin. Oncol..

[23] Ahmad S., Fergus K., McCarthy M. (2015). Psychosocial issues experienced by young women with breast cancer: The minority group with the majority of need. Curr. Opin. Supportive Palliat. Care.

[24] Bidstrup P.E., Christensen J., Mertz B.G., Rottmann N., Dalton S.O., Johansen C. (2015). Trajectories of distress, anxiety, and depression among women with breast cancer: Looking beyond the mean. Acta Oncol. (Stock. Swed.).

[25] Kramer J.R., Ledolter J., Manos G.N., Bayless M.L. (2000). Stress and metabolic control in diabetes mellitus: Methodological issues and an illustrative analysis. Ann. Behav. Med. A Publ. Soc. Behav. Med..

[26] Parks C.G., Miller D.B., McCanlies E.C., Cawthon R.M., Andrew M.E., DeRoo L.A., Sandler D.P. (2009). Telomere length, current perceived stress, and urinary stress hormones in women. Cancer Epidemiol. Biomark. Prev. Publ. Am. Assoc. Cancer Res. Cosponsored Am. Soc. Prev. Oncol..

[27] Waldman S.V., Diez J.C., Arazi H.C., Linetzky B., Guinjoan S., Grancelli H. (2009). Burnout, perceived stress, and depression among cardiology residents in Argentina. Acad. Psychiatry J. Am. Assoc. Dir. Psychiatr. Resid. Train. Assoc. Acad. Psychiatry.

[28] Herschbach P., Keller M., Knight L., Brandl T., Huber B., Henrich G., Marten-Mittag B. (2004). Psychological problems of cancer patients: A cancer distress screening with a cancer-specific questionnaire. Br. J. Cancer.

[29] Thewes B., Butow P., Girgis A., Pendlebury S. (2004). The psychosocial needs of breast cancer survivors; a qualitative study of the shared and unique needs of younger versus older survivors. Psychooncology.

[30] Bond M.H. (2010). Oxford Handbook of Chinese Psychology.

[31] Wong P.T.P., Wong L.C.J. (2006). Handbook of Multicultural Perspectives on Stress and Coping.

[32] Chuang W.L., Chin C.C. (2002). Information Needs of a Woman Newly Diagnosed with Breast Cancer. J. Heal. Sci..

[33] Lee T.Y., Chen H.H., Yeh M.L., Li H.L., Chou K.R. (2013). Measuring reliability and validity of a newly developed stress instrument: Newly Diagnosed Breast Cancer Stress Scale. J. Clin. Nurs..

[34] Li X., Li Y., Han N., Chen Y. (2018). Reliability and validity analysis of Newly Diagnosed Breast Cancer Stress Scale. Chin. J. Mod. Nurs..

[35] ΜAΡΙA Χ. (2019). Στάθμιση του ερωτηματολογίου “ Κλίμακα Στρες σε Πρόσφατα Διαγνωσμένες με Καρκίνο του Μαστού [Stress Management and Health Promotion Intervention on Breast Cancer Patients].

[36] Li X.L., Wang J.H., He S.L. (2018). Confirmatory factor analysis of the shortened dentine hypersensitivity experience questionnaire. Hua Xi Kou Qiang Yi Xue Za Zhi = Huaxi Kouqiang Yixue Zazhi West China J. Stomatol..

[37] Kline R.B. (2005). Principles and Practice of Structural Equation Modeling.

[38] Bentler P.M. (1982). Confirmatory Factor Analysis via Noniterative Estimation: A Fast, Inexpensive Method. J. Mark. Res..

[39] Doll W.J., Xia W., Torkzadeh G. (1994). A confirmatory factor analysis of the end-user computing satisfaction instrument. MIS Q..

[40] MacCallum R.C., Hong S. (1997). Power Analysis in Covariance Structure Modeling Using GFI and AGFI. Multivar. Behav. Res..

[41] Effendi I., Chaniago S., Lestari I., Nasib N., Chaniago S., Azzahra A.s. (2019). Trust identification and smartphone purchase decisions (Structural equation modeling approach). Int. J. Civ. Eng. Technol..

[42] Hu L.t., Bentler P.M. (1999). Cutoff criteria for fit indexes in covariance structure analysis: Conventional criteria versus new alternatives. Struct. Equ. Modeling Multidiscip. J..

[43] McDonald R.P., Ho M.H. (2002). Principles and practice in reporting structural equation analyses. Psychol. Methods.

[44] Luo B., Xiao S. (2019). Reliability and validity for Chinese version of the 9-item Shared Decision Making Questionnaire. Zhong Nan Da Xue Xue Bao. Yi Xue Ban J. Cent. South Univ. Med Sci..

[45] Mulaik S.A., James L.R., Van Alstine J., Bennett N., Lind S., Stilwell C.D. (1989). Evaluation of goodness-of-fit indices for structural equation models. Psychol. Bull..

[46] Breivik E., Olsson U.H. (2001). Adding Variables to Improve Fit: The Effect of Model Size on Fit Assessment in LISREL.

[47] Abdollahi A., Panahipour H., Hosseinian S., Allen K.A. (2019). The effects of perceived stress on hope in women with breast cancer and the role of psychological hardiness. Psychooncology.

[48] Andrykowski M.A., Cordova M.J., Studts J.L., Miller T.W. (1998). Posttraumatic stress disorder after treatment for breast cancer: Prevalence of diagnosis and use of the PTSD Checklist-Civilian Version (PCL-C) as a screening instrument. J. Consult. Clin. Psychol..

[49] Cohen S., Kamarck T., Mermelstein R. (1983). A Global Measure of Perceived Stress. J. Health Soc. Behav..

[50] McDonald S.D., Beckham J.C., Morey R.A., Calhoun P.S. (2009). The validity and diagnostic efficiency of the Davidson Trauma Scale in military veterans who have served since September 11th, 2001. J. Anxiety Disord..

[51] Rochette A., Bravo G., Desrosiers J., St-Cyr Tribble D., Bourget A. (2007). Adaptation process, participation and depression over six months in first-stroke individuals and spouses. Clin. Rehabil..

[52] Andersen K.G., Kehlet H. (2011). Persistent pain after breast cancer treatment: A critical review of risk factors and strategies for prevention. J. Pain.

[53] Bodtcher H., Bidstrup P.E., Andersen I., Christensen J., Mertz B.G., Johansen C., Dalton S.O. (2015). Fatigue trajectories during the first 8 months after breast cancer diagnosis. Qual. Life Res..

[54] Hidding J.T., Beurskens C.H., van der Wees P.J., van Laarhoven H.W., Nijhuis-van der Sanden M.W. (2014). Treatment related impairments in arm and shoulder in patients with breast cancer: A systematic review. PLoS ONE.

[55] Mertz B.G., Bistrup P.E., Johansen C., Dalton S.O., Deltour I., Kehlet H., Kroman N. (2012). Psychological distress among women with newly diagnosed breast cancer. Eur. J. Oncol. Nurs..

[56] Mitchell A.J., Ferguson D.W., Gill J., Paul J., Symonds P. (2013). Depression and anxiety in long-term cancer survivors compared with spouses and healthy controls: A systematic review and meta-analysis. Lancet Oncol..

[57] Mertz B.G., Dunn-Henriksen A.K., Kroman N., Johansen C., Andersen K.G., Andersson M., Mathiesen U.B., Vibe-Petersen J., Dalton S.O., Envold Bidstrup P. (2017). The effects of individually tailored nurse navigation for patients with newly diagnosed breast cancer: A randomized pilot study. Acta Oncol. (Stockh. Swed.).

[58] Livsey K.R., Rossitch J.C., Tait E., Mannle S.E., Smith R. (2017). Where Fear Begins: The Effect of a Nurse Navigator Home Visit to Decrease Distress in Newly Diagnosed Breast Cancer Patients. J. Oncol. Navig. Surviv..

[59] Li P., Guo Y.J., Tang Q., Yang L. (2018). Effectiveness of nursing intervention for increasing hope in patients with cancer: A meta-analysis. Rev. Lat. Am. Enferm..

