# Supplementary material for: An Improved Stress-Scale Specifically Designed to Measure Stress of Women with Newly Diagnosed Breast Cancer
Source: Int J Environ Res Public Health. 2021 Feb 27;18(5):2346. doi: 10.3390/ijerph18052346 (PMC7967728; doi:10.3390/ijerph18052346)
Supplement: Supplementary file 1 [file ijerph-18-02346-s001.pdf]

Supplementary Information. Item scores of NDBCSS-R.

Content	scores<br>Mean ± SD
1. I often cry	3.25 ± 0.75
2. Illness makes me worry about my family	3.28 ± 0.72
3. Loss of the breast will affect my life	2.74 ± 0.86
4. I have fear, anxiety and depression	2.12 ± 0.81
5. Illness makes me worry about my job	2.46 ± 0.92
6. I cannot make decision for my breast cancer treatment	3.03 ± 0.83
7. I think the road of anti-cancer is lonely, hard and there is a lack of support	2.36 ± 0.94
8. I am worried that my arm cannot lift heavy weight and it will affect my life and work	2.15 ± 0.90
9. I am worried that my economic conditions cannot deal with the required medical expenses	3.14 ± 0.80
10. I am very worried about the uncertainty of the progression of illness	2.32 ± 0.95
11. I am worried about the side effects caused by chemotherapy: such as physical discomfort, change of appearance, or future birth plans, etc.	2.32 ± 0.81
12. Loss of the breast will affect my attractiveness to my partner	2.70 ± 0.87
13. Insufficient breast cancer information scares me	2.37 ± 0.91
14. I can accept the staging of breast cancer	2.98 ± 0.85
15. I can accept the diagnosis of breast cancer	3.13 ± 0.77
16. I can able to make proper arrangements and deal with things affected by illness	3.05 ± 0.68
17. I use some adaptation methods to face cancer	3.13 ± 0.64
